# Punicalagin Decreases Serum Glucose Levels and Increases PON1 Activity and HDL Anti-Inflammatory Values in Balb/c Mice Fed a High-Fat Diet

**DOI:** 10.1155/2018/2673076

**Published:** 2018-07-31

**Authors:** Dana Atrahimovich, Abraham O. Samson, Ali Khattib, Jacob Vaya, Soliman Khatib

**Affiliations:** ^1^Department of Oxidative Stress and Human Diseases, MIGAL-Galilee Research Institute, 11016 Kiryat Shmona, Israel; ^2^Tel-Hai College, 12208 Upper Galilee, Israel; ^3^Faculty of Medicine in the Galilee, Bar-Ilan University, 1311502 Safed, Israel

## Abstract

Polyphenols are consumed daily in the human diet and are associated with reduced risk of a number of chronic diseases, including cancer, cardiovascular disease, and diabetes. Traditionally, the health benefits of polyphenols have been attributed to their antioxidant activity, but many studies might be hampered by oral administration and insignificant bioavailability. Rather than exerting a direct antioxidant effect, the mechanisms by which polyphenols express their beneficial effect seem to involve their interaction with proteins. The present study is aimed at broadening and confirming our recently published in vitro results showing that polyphenols may reduce atherosclerosis risk via interaction with proteins and lipoproteins related to atherosclerosis. The biological functions of punicalagin and quercetin in relation to glucose and lipid levels, paraoxonase 1 (PON1) activity, and inflammation were examined in vivo. Mice were fed a high-fat diet (HFD) for 12 weeks, and during the last 4 weeks, they received subcutaneous treatments via implanted minipumps, which released physiological concentrations of punicalagin, quercetin, or atorvastatin (as a positive control) daily into the serum. The HFD reduced serum PON1 activity, whereas punicalagin administration restored PON1 activity to the level of mice fed a normal diet. In addition, punicalagin significantly reduced glucose levels in HFD mice and improved HDL anti-inflammatory properties. In conclusion, beyond antioxidant activity, the mechanisms by which polyphenols exert their beneficial properties appear to involve their interaction with serum proteins that mediate HDL function and lipid-glucose state in the circulation.

## 1. Introduction

Polyphenols are abundant antioxidants in the human diet, found in fruits, vegetables, nuts, and tea. They have been proposed to exert beneficial effects in a multitude of disease states, including cancer, cardiovascular disease, and neurodegenerative disorders [1, 2]. Quercetin, a polyphenol found mostly in red onion *(Allium cepa)*, and punicalagin, from pomegranate *(Punica granatum)*, are two prominent antioxidants in the human diet. Beyond their antioxidant, chemopreventive, and antibacterial properties, quercetin and punicalagin are known for their hypoglycemic and antiatherogenic activities [1, 3–5]. Both polyphenols have been shown to protect macrophage cells from lipid accumulation and foam cell formation and to reduce the development of atherosclerosis [4, 6–9]. In addition, numerous studies have demonstrated that quercetin and its glycosides, as well as the antioxidant composition of pomegranate—especially total phenolics and total anthocyanin content, are effective in the prevention and treatment of noninfectious chronic diseases such as diabetes, obesity, and hyperlipidemia. They can regulate glucose and lipid metabolism through different mechanisms. They decrease blood glucose by protecting pancreatic *β*-cells, improving insulin sensitivity, or both. They also reduce lipid levels, possibly through regulation of lipid catabolism and/or anabolism [10, 11]. Polyphenols' mechanism of action is assumed to stem from their antioxidant activity, driven by their chemical property of donating an electron or chelating transition metals. However, antioxidant activity cannot be the sole explanation for polyphenols' cellular effects in vivo since they are poorly absorbed through the gut into the bloodstream and extensively metabolized in the small intestine, liver, and colon. Thus, concentrations of polyphenols in the blood scarcely reach the level needed for effective antioxidant activity [12]. Recent studies have suggested that the cellular effects of polyphenols may be mediated by their interaction with specific proteins. Polyphenols interact selectively with different components of proteins and intracellular enzymes (e.g., hydrolases, oxidases, and kinases) [13–17], serum enzymes such as albumin or amylase, estrogen receptors, and various transcription factors [16, 18, 19]. Similarly, polyphenols have been found to act as ligands of nuclear receptors, proliferate, activate, and modulate energy and homeostasis [12].

Low-density lipoprotein (LDL) is an important carrier of cholesterol in the body. LDL contains one large protein molecule—apolipoprotein B-100—that is recognized and bound by the LDL receptor. Epidemiological data reveal that the more LDL is present in the blood, the more rapidly atherosclerosis develops [20]. In contrast, high-density lipoprotein (HDL) plays an important role in preventing atherosclerosis due to its antiatherogenic properties, such as reverse cholesterol transport, antioxidant, anti-inflammatory, antiapoptotic and vasodilatory activities, and endothelial function improvement [21, 22]. Most of the atheroprotective effects of HDL are attributed to its associated enzyme paraoxonase 1 (PON1; EC 3.1.8.1) [23, 24]. PON1-deficient mice are susceptible to the development of atherosclerosis, whereas it is inhibited by overexpression of human PON1 in mice [25]. PON1 activity has been inversely correlated with carotid intima-media thickness [26], attenuates oxidized-LDL uptake by macrophages, inhibits macrophage cholesterol biosynthesis rate, and stimulates HDL-mediated cholesterol efflux from macrophages [27–30]. Epidemiological evidence demonstrates the association between low PON1 activity and increased risk of cardiovascular events [26, 31]. In addition, reports from studies with children and adolescents from different ethnic groups have shown lower PON1 activity associated with type 2 and type 1 diabetes [32–37]. Diabetes and atherosclerosis are two diseases that are known to occur simultaneously, with atherosclerosis being accelerated by diabetes and metabolic syndrome [38]. In vitro studies have provided important clues to the mechanism by which hyperglycemia might lead to atherosclerosis, but these mechanisms have not always been borne out in vivo. Thus, many studies have been conducted to link a high-glucose-level state with HDL, PON1, and atherosclerosis.

In previous studies, we have shown that polyphenols, such as quercetin, bind to an allosteric site on recombinant PON1 and affect the enzyme's function and biology [17]. Polyphenols prevent PON1 oxidation and protect it from linoleic acid hydroperoxide inhibition via specific interaction [16]. Similarly, punicalagin binds to apolipoprotein B-100 and induces LDL influx into macrophages to a level that prevents their transformation into foam cells. Such an effect might provide an alternative mechanism for lowering blood cholesterol concentration and attenuating the development of atherosclerosis [39].

To validate our recent in vitro results showing antiatherogenic effects on punicalagin and quercetin upon specific binding to a protein located on HDL or LDL particles [39], we aimed to characterize those polyphenols' effects in vivo. In this study, the effects of punicalagin and quercetin, administered subcutaneously (sc) via osmotic minipumps, were investigated in mice fed a high-fat diet (HFD). Minipump implantation allowed us to monitor lipid and glucose levels in HFD mouse serum in the presence of physiological concentrations of the polyphenols and to measure HDL and PON1 properties at the end of the experiment.

## 2. Materials and Methods

### 2.1. Materials

Punicalagin, quercetin, Tween 80, methanol, chloroform, atorvastatin, dihydrocoumarin, dimethyl sulfoxide (DMSO), 1-palmitoyl-2-arachidonoyl-*sn*-glycero-3-phosphorylcholine (PAPC), and dichloro-dihydro-fluorescein diacetate (DCFH-DA) were purchased from Sigma-Aldrich; 1-palmitoyl-2-(5,6-epoxyisoprostane E2)-*sn*-glycero-3-phosphocholine (PEIPC) was prepared from PAPC, and ALZET osmotic minipumps were purchased from Biotest.

### 2.2. Animals and Diets

#### 2.2.1. Animals

Altogether, 90 male Balb/c mice that were 8 weeks old at the start of the experiment were taken for this study, out of which 68 animals survived until sacrifice. Animals were purchased from Harlan Laboratories, Israel, and were housed in groups of 5-6 in 26.5 × 20 × 13.5 cm cages in the SPF-certified facility of the Sharett Institute, Hadassah-Hebrew University Medical Center. Food and water were provided ad libitum. Mice were kept under a 12 h light-dark cycle (lights on at 0700 h). Ethical approval for this research was provided by the Authority for Biological and Biomedical Models, Hebrew University of Jerusalem, MD-16-14817-4.

#### 2.2.2. Study Design

The objective of this research was to examine the effects of polyphenols on lipid metabolism, lipoprotein biology, and atherosclerosis in HFD-fed mice.

The initial body weights (BWs) were measured, and blood samples (~0.1 ml) were collected to determine baseline serum lipid levels following a 12 h fast. Blood was collected from the facial vein (under anesthesia). Mice were divided into 2 groups. The control (nongroup) (*n* = 18 at protocol initiation) was fed a normal rodent diet for 8 weeks, while the remaining groups (*n* = 72 at protocol initiation) were fed a HFD (60 cal% fat, 20 cal% proteins, and 20 cal% carbohydrates; D12492, Research Diets Inc., USA). After 8 weeks and a further 12 h fast, the animals were weighed and blood was collected to determine serum lipid levels. The mice were fed normal (nongroup) or HFD (treatment groups) for another 4 weeks. During this time, the treatment groups (*n* = 18 each) received the following treatments administered via sc-implanted osmotic minipumps: (1) vehicle (double-distilled water (DDW) + 2% Tween 80), (2) punicalagin at 140 *μ*g/100 *μ*l, (3) quercetin at 42 *μ*g/100 *μ*l, and (4) atorvastatin at 15 mg/100 *μ*l. All compounds were first dissolved in ethanol, which was evaporated under nitrogen stream, and the residue was dissolved in DDW + 2% Tween 80 before use, as recommended by the ALZET minipump supplier. Each pump was loaded with 100 *μ*l of the solution (molecule in DDW + 2% Tween 80) from which 3.5 *μ*l, equivalent to 2 *μ*M, was released daily for 28 days. Pumps were implanted under a cocktail of Domitor plus ketamine injected intraperitoneally (ip). The depth of the anesthesia was monitored by a toe pinch reflex test. The area of implantation was shaved, disinfected with 70% ethanol, and wiped with iodine using a swab stick or pads. Each minipump was inserted sc into the interscapular space. The skin was then stitched, and the animal allowed to recover. A half hour before the surgery, mice were administered 5 mg/kg of the analgesic Rimadyl. Animals were observed and monitored daily for discomfort, ulcerations, and immobility. Mice suffering from these discomforts were immediately euthanized.

At the end of the treatment period and following 12 h of fasting, the mice were sacrificed by ip injection of veterinary pental and intracardial blood was collected for end-of-protocol measurements of the relevant blood parameters.

### 2.3. Serum Samples

Blood drawn during animal sacrifice was collected in conical Eppendorf tubes and allowed to clot for half an hour. Then, the blood samples were centrifuged at 2000 rpm for 5 min. The serum was stored at −70°C until analysis. The following indicators of hyperlipidemia and atherosclerosis were measured: serum lipid levels (HDL, triglycerides, and total cholesterol) and glucose levels.

#### 2.3.1. PON1 Lactonase Activity

A sample of mouse serum diluted 10-fold with phosphate-buffered saline (PBS) was placed in a 96-well UV microplate containing 45 *μ*l PBS and 50 *μ*l Tris-HCl buffer pH 8.4 with 1 mM CaCl_2_ (activity buffer) per well. Then, 100 *μ*l dihydrocoumarin, 2 mM in the activity buffer, was added (prepared from a stock of 100 mM dihydrocoumarin in DMSO). Dihydrocoumarin hydrolysis rate was measured at 270 nm every 25 s for 15 min using a SpectraMax M2 reader. Nonenzymatic hydrolysis of dihydrocoumarin was subtracted from the total rate of hydrolysis. One unit of lactonase activity was equal to the hydrolysis of 1 *μ*mol dihydrocoumarin per minute.

#### 2.3.2. Anti-Inflammatory Activity Measurements Using Dichlorofluorescein (DCF) Cell-Free Assay (CFA)

HDL was isolated from mouse serum with the LDL/VLDL and HDL Purification Kit (Cell Biolabs, catalog number STA-608) and dialyzed twice, for 1 h each time, and once more overnight against PBS at 4°C. To prepare PEIPC by oxidation, 1 mg of PAPC in 100 *μ*l chloroform was evaporated under a nitrogen stream. The lipid residue was allowed to autoxidize under exposure to air for 24–48 h. PEIPC was dissolved in 400 *μ*l DMSO and identified by LC-MS [40]. DCFH-DA was dissolved in methanol to prepare a stock solution (2 mg/ml). Prior to each experiment, 50 *μ*l of the stock was added to 200 *μ*l of 0.1 M NaOH and incubated for 30 min at room temperature in the dark. The reaction was terminated by neutralizing the solution with 1.75 ml of 0.1 M PBS, resulting in the conversion of DCFH-DA to dihydrodichlorofluorescein (DCFH). Upon oxidation, DCFH transforms into DCF. HDL (73 *μ*l) was incubated at a final concentration of 50 *μ*g with 2 *μ*l PEIPC (50 *μ*g/ml) in a black flat-bottom ELISA plate at 37°C for 60 min. Then, 25 *μ*l DCFH solution (50 *μ*g/ml) was added to each well, mixed, and incubated at 37°C for 3 h. Fluorescence intensity was determined using a Tecan Infinite® 200 PRO Plate Reader with an excitation wavelength of 485 nm and emission wavelength of 530 nm. Fluorescence in the absence of HDL was normalized to 1.0. Values > 1.0 after addition of the test HDL indicated proinflammatory HDL; values < 1.0 indicated anti-inflammatory HDL.

### 2.4. Statistical Analysis

Statistical analysis was carried out using GraphPad Prism 5.01 software. Student's paired *t*-test was used to compare the means of two groups with significance determined at *P* < 0.05 (∗), *P* < 0.01(∗∗), or *P* < 0.001 (∗∗∗).

## 3. Results

We have recently shown that punicalagin binds specifically to LDL and induces its influx into macrophage cells without foam cell formation. Such a mechanism may remove excess cholesterol from these cells to the liver, thereby lowering cholesterol levels in the circulation [39]. In this study, the antiatherogenic and anti-inflammatory properties of punicalagin and its ability to affect serum lipid and glucose levels were explored in vivo.

### 3.1. BW and Clinical Parameters in Blood Samples

**Figure 1:**
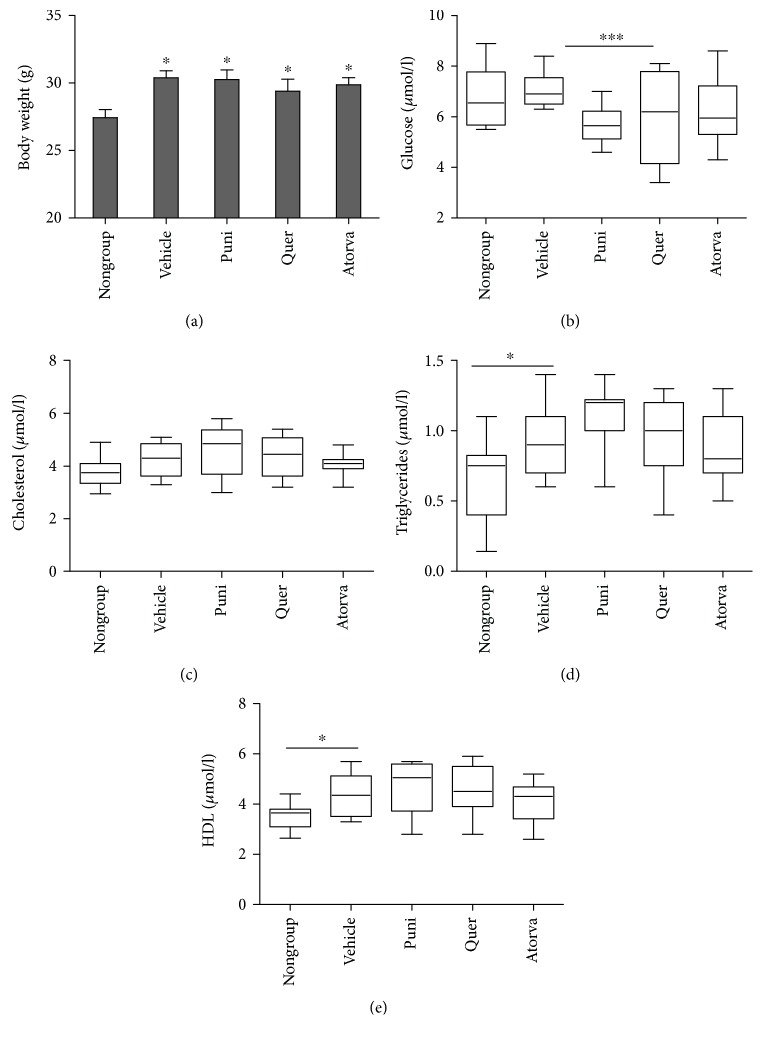
Effect of sc-implanted pump-administered punicalagin (puni) at 70 *μ*g/kg BW, quercetin (quer) at 140 *μ*g/kg BW, or atorvastatin (atorva) at 15 mg/kg BW on body weight (a), and glucose (b), cholesterol (c), triglyceride (d), and HDL (e) levels in the sera of mice sacrificed at the end of the treatment period. Each box represents mean ± SEM for 11–13 mice. Control group (vehicle) consisted of mice fed a HFD and administered DDW + 2% Tween 80. Nongroup mice were fed a regular diet. ^∗^*P* < 0.05, ^∗∗∗^*P* < 0.001.

Mouse BW and blood samples were taken three times during the experiment: at its initiation, midpoint, and termination. Final BW at the end of the experiment is reported in Figure 1(a). Significant changes in BW were observed for all groups of mice fed a HFD (experimental groups compared to nongroup, *P* < 0.05). Mouse BW was not affected by sc-implanted pump treatments. Levels of glucose, triglycerides, cholesterol, and HDL (Figure 1) were determined from serum taken when the mice were sacrificed. The four experimental groups fed a HFD were treated sc with osmotic minipumps loaded as follows: DDW + 2% Tween 80 (vehicle), punicalagin (70 *μ*g/kg BW), quercetin (140 *μ*g/kg BW), or atorvastatin (15 mg/kg BW). Serum values of glucose, triglycerides, cholesterol, and HDL upon treatment administration were compared to the control group (normal diet-fed mice) which was not treated sc (nongroup). An interesting finding can be seen in Figure 1(b); administration of punicalagin significantly decreased serum glucose levels in comparison to the vehicle group.  Figure 1(d) shows that although the HFD led to increased triglyceride levels (*P* < 0.05), there was no effect of the treatments on serum triglyceride levels. Atorvastatin is a known cholesterol-lowering drug that inhibits cell cholesterol synthesis [41]. In this study, it was used as a positive control and although its effects were not statistically significant, it reduced total cholesterol and HDL in the serum (Figures 1(c) and 1(e), respectively).  Figure 1(c) shows an upward trend (not significant) in total cholesterol level upon saline, punicalagin, and quercetin administration in comparison to the control (nongroup). HDL levels increased with administration of a HFD (*P* < 0.05) between nongroup and vehicle group, Figure 1(e), but neither punicalagin nor quercetin treatments changed HDL levels (Figure 1(e)).

### 3.2. PON1 Activity

**Figure 2:**
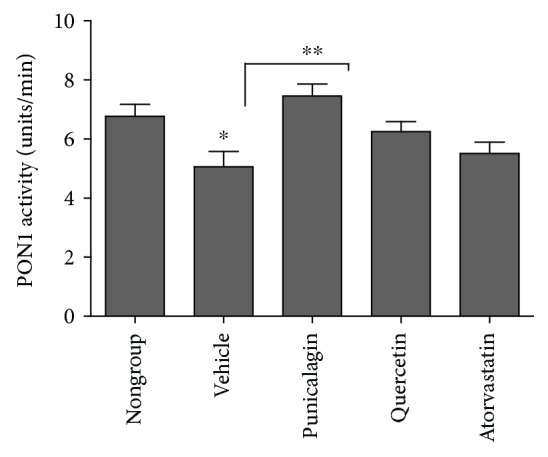
PON1 lactonase activity in the sera of mice sacrificed at the end of the treatment period. Each column represents mean ± SEM for 11–13 mice. Control group (vehicle) consisted of mice fed a HFD and administered DDW + 2% Tween 80. Nongroup mice were fed a regular diet. ^∗^*P* < 0.05, ^∗∗^*P* < 0.01.

Serum PON1 activity is shown in Figure 2. PON1 activity was reduced in the sera of HFD-fed mice (vehicle compared to nongroup, *P* < 0.05), and punicalagin restored PON1 activity (to nongroup levels; approximately 8 units/min compared to 5 units/min for the vehicle group, *P* < 0.01).

### 3.3. Anti-Inflammatory Properties of HDL

**Figure 3:**
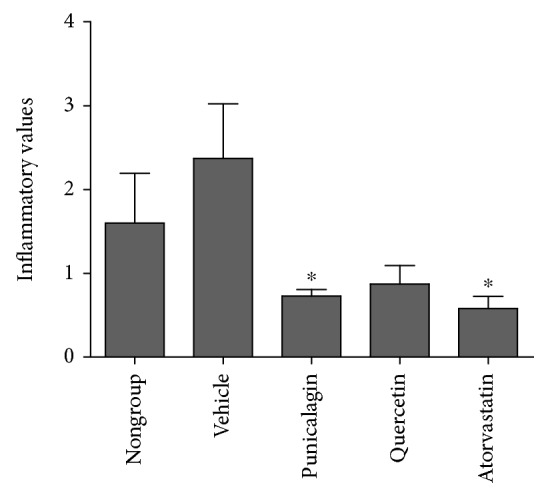
Anti-inflammatory activity of HDL. Values are presented as inflammatory/anti-inflammatory properties of HDL isolated from the sera of the treated mice. Fluorescence in the absence of HDL was normalized to 1.0. Values > 1.0 after addition of the test HDL indicated proinflammatory activity; values < 1.0 indicated anti-inflammatory activity. Control group (vehicle) consisted of mice fed a HFD and administered DDW + 2% Tween 80. Nongroup mice were fed a regular diet. ^∗^*P* < 0.05 relative to vehicle.

The CFA method detects dysfunctional HDL by revealing results comparable to those of a cell-based assay [20, 42]. The CFA is based on HDL's ability to prevent oxidation of DCFH by oxidized lipids, which leads to conversion of the nonfluorescent DCFH to its fluorescent form, DCF. DCFH was oxidized by PEIPC since this oxidized phospholipid accounts for more than 80% of the LDL-induced monocyte chemotactic activity in human artery wall cell cocultures (i.e., 80% of the monocyte chemotactic activity resulting from the addition of LDL to the cocultures is attributable to the formation of PEIPC) [43].  Figure 3 shows that HDL from the HFD mice treated with vehicle was twice as proinflammatory as that from mice fed a regular diet. HDL isolated from mice treated with punicalagin had a significant anti-inflammatory effect (value < 1) as compared to the vehicle group. As for quercetin, its anti-inflammatory value was approximately 1.0 which, compared to the vehicle group, was not significant.

## 4. Discussion

Polyphenols have been intensively explored for their antiatherogenic and hypoglycemic properties [4]. With respect to atherosclerosis, polyphenols have been shown to reduce serum LDL modifications, macrophage lipid peroxidation, cholesterol synthesis, atherosclerotic lesion area, and foam cell formation [21, 44]. As for hypoglycemia, significant evidence suggests that polyphenol-rich diets have the ability to protect against diabetes [45, 46]. Although the health beneficial capabilities of both quercetin and punicalagin have been well studied, much uncertainty surrounds the underlying mechanisms of action. There is an overall tendency to explain the antiatherogenic effects of a polyphenol by its antioxidant activity, even though polyphenol concentrations in the blood barely reach the level required for such activity (10–100 *μ*M) [12]. Recent studies have suggested that the cellular effects of polyphenols are mediated by their interaction with specific intracellular elements or plasma proteins [19]. Studies in our laboratory have shown that binding of the polyphenol glabridin to PON1 prevents the enzyme's inhibition by linoleic acid hydroperoxide [16]. Moreover, punicalagin, but not quercetin or other polyphenols, binds to LDL and induces its influx into macrophages without foam cell formation. This could be proposed as a mechanism that lowers cholesterol blood concentration and attenuates the development of atherosclerosis [39]. The present study is aimed at supporting this latter finding and to examine the biological functions of punicalagin and quercetin in relation to glucose and lipid levels, PON1 activity, and inflammation in the sera of hyperlipidemic mice. Mice were fed a HFD for 12 weeks, and during the last 4 weeks, they also received treatments via minipump, which released physiological concentrations of each molecule daily for 28 days. It was important to keep the daily polyphenol doses in the mouse serum at no more than 2 *μ*M, which is close to the approximate serum concentration of polyphenols in the human blood [47, 48] and is below the concentration at which polyphenols have been shown to act through their classical antioxidant activity mechanism [12, 49, 50]. At the end of the treatment period, the mice were sacrificed and clinical parameters were measured, quantified, and compared (Figure 1). As polyphenols are extensively metabolized in the small intestine, liver, and colon [51, 52], they were administered into the circulation via osmotic minipumps implanted sc, ensuring exposure to the test molecules at predictable times each day without repetitive injection schedules. This method protects the mice from excessive stress and provides consistent and reliable results. Moreover, the sc alternative is better than the digestive system alternative as polyphenols are extensively catabolized in the small intestine, liver, and colon [12], making it difficult to control their serum concentration with dietary administration. This is the first time that this type of administration has been used for polyphenols, and it allowed us to accurately determine their effects on glucose and lipid levels and on inflammatory state and antiatherogenic PON1 protein levels in vivo.

The studied polyphenols had no significant effect on HDL, cholesterol, or triglyceride levels. This finding is in line with the fact that among the plethora of in vitro and in vivo studies related to punicalagin and quercetin, very few show any effect on serum LDL, HDL, total cholesterol, or triglyceride levels. Most of the studies highlight additional antiatherogenic parameters of these polyphenols, such as their ability to attenuate macrophage cholesterol accumulation, reduce carotid thickness, decrease the number of foam cells in lesions, and reduce LDL modifications [5–7, 9, 17, 44, 53, 54]. Recently, research has focused on HDL quality in the circulation rather than its quantity. HDL is classified into subclasses that differ in density, size, content, and electrophoretic mobility, and its heterogeneity affects its function [55]. For example, structure-function analysis has shown that HDL's atheroprotective functions are predominantly associated with the smaller, denser, and protein-rich HDL [22]. PON1 is a most important enzyme bound to antioxidant and antiatherogenic HDL, responsible for many of HDL's benefits; correlations between PON1, HDL, and atherosclerosis have been well established, both in vivo and in vitro [26, 56]. Epidemiological evidence demonstrates that low PON1 activity is associated with increased risk of cardiovascular events and cardiovascular disease [29, 34]. Here, both quercetin and punicalagin increased the activity of PON1 (only punicalagin's effect was significant) (Figure 2).

The benefits of dietary polyphenols for type 2 diabetes are protection of pancreatic *β*-cells against glucose toxicity, anti-inflammatory and antioxidant effects, inhibition of *α*-amylases or *α*-glucosidases and thus decrease of starch digestion, and inhibition of advanced glycation end-product formation [45]. A recent study has suggested that supplementation with pomegranate peels leads to increased insulin levels [57]. Therefore, our finding that administration of punicalagin to HFD mice significantly decreases their serum glucose levels (*P* < 0.001, Figure 1(b)) is highly important. We found a correlation between serum PON1 lactonase activity and serum glucose concentration: the latter increased in HFD-fed mice relative to the nongroup, whereas their PON1 lactonase activity decreased significantly. Interestingly, the group treated with punicalagin showed decreased serum glucose concentrations that paralleled increasing serum lactonase activity (both statistically significant), similar to the nongroup. These findings demonstrate a correlation between PON1 lactonase activity and serum glucose levels that warrants further investigation, particularly in light of the recent reports from studies with different ethnic groups that have shown reduced PON1 activities associated with diabetes [32–37].

The anti-inflammatory potential of punicalagin and quercetin has been well-studied [58, 59]. Although in this study, quercetin's anti-inflammatory activity was not statistically significant, it did manifest a highly effective anti-inflammatory property. Quercetin's known anti-inflammatory potential is expressed with different cell types, in both animal and human models. It also plays a modulating, biphasic, and regulatory role in inflammation and immunity [1]. Punicalagin, however, showed a significant anti-inflammatory effect for HDL isolated from the sera of mice administered punicalagin via minipump. In addition, HDL isolated from mice treated with atorvastatin showed a significant anti-inflammatory effect, which was expected [41].

The present study shows that although HDL level did not decrease upon punicalagin administration, its biological activity (quality) was improved, as reflected by its anti-inflammatory properties and activity of the PON1 bound to it. Sc administration of punicalagin to HFD-fed mice simultaneously reduced the glucose level in the circulation and increased PON1 activity.

We suggest an additional mechanism for polyphenols' beneficial effects on HDL, beyond the classically proposed antioxidant activity. As the hypoglycemic, anti-inflammatory, and antiatherogenic effects were demonstrated at small, physiological concentrations of polyphenols, we suggest that rather than exerting direct antioxidant effects, the mechanisms by which polyphenols express these beneficial properties involve their interaction with cellular signaling pathways and cell and serum proteins that mediate cell function. Further investigation of the effects of polyphenols on HDLs' chemical and biological activity and their underlying mechanisms is therefore warranted.

## Data Availability

The data used to support the findings of this study are available from the corresponding author upon request.

## Abbreviations

LDL: Low-density lipoprotein
HDL: High-density lipoprotein
PON1: Paraoxonase 1
sc: Subcutaneous
DMSO: Dimethyl sulfoxide
PAPC: 1-Palmitoyl-2-arachidonoyl-*sn*-glycero-3-phosphorylcholine
DCFH-DA: Dichloro-dihydro-fluorescein diacetate
PEIPC: 1-Palmitoyl-2-(5,6-epoxyisoprostane E2)-*sn*-glycero-3-phosphocholine
BW: Body weight
ip: Intraperitoneal
PBS: Phosphate-buffered saline
DCF: Dichlorofluorescein
CFA: Cell-free assay
DCFH: Dihydrodichlorofluorescein
HFD: High-fat diet.
